# Protocol of End-PSCI trial: a multicenter, randomized controlled trial to evaluate the effects of DL-3-n-butylphthalide on delayed-onset post stroke cognitive impairment

**DOI:** 10.1186/s12883-022-02957-y

**Published:** 2022-11-16

**Authors:** Ziyu Liu, Wenhui Lu, Ling Gao, Xiaojuan Guo, Jie Liu, Fan Gao, Kang Huo, Jin Wang, Qiumin Qu

**Affiliations:** 1 Department of Neurology, The First Affiliated Hospital of Xi’an Jiaotong University, 277 West Yanta Rd, Xi’an, 710061 China; 2 Clinical research center, The First Affiliated Hospital of Xi’an Jiaotong University, Xi’an, China; 3 Center for Brain Science, The First Affiliated Hospital of Xi’an Jiaotong University, Xi’an, China

**Keywords:** DL-3-n-butylphthalide, Post stoke cognitive impairment, Prevention, White matter degeneration

## Abstract

**Background:**

Delayed-onset post stroke cognitive impairment (PSCI) results from secondary neurodegeneration induced by stroke. Whereas targeted prevention or treatment strategies are still missing due to lack of evidences. This trial aims to evaluate the preventive effects of DL-3-n-butylphthalide (NBP) on delayed-onset PSCI.

**Methods:**

Effects of NBP on Delayed-onset Post Stroke Cognitive Impairment (End-PSCI) is a prospective, parallel-group, open-label, multicenter, randomized controlled trial with blinded outcome assessment. Hospital patients with acute cerebral infarction (within 2 weeks of onset) will be randomized into either standard medical therapy group or standard medical therapy combined NBP treatment group (NBP 200 mg, three times per day for 24 weeks). The primary outcome is the difference of incidence of delayed-onset PSCI between two groups. The secondary outcomes include difference of white matter degeneration, cognitive scores and prevalence of early-onset PSCI between two groups.

**Discussion:**

End-PSCI trial will provide evidences for NBP preventing delayed-onset PSCI. The secondary outcomes will also provide valuable insights into the pathogenesis of delayed-onset PSCI and mechanism of NBP’s actions.

**Trial registration:**

Trialsearch.who.int, ChiCTR2000032555, 2020/5/2, prospectively registered.

## Background

Post stroke cognitive impairment (PSCI) indicates cognitive impairment resulting from a stroke event, including post stroke dementia (PSD) and PSCI not fulfilling criteria for dementia [1]. Most PSCI occur in the early stage after stroke (early-onset PSCI), while some patients without early-onset PSCI will develop cognitive impairment months after stroke (delayed-onset PSCI) [2–5]. Data of the prevalence of delayed-onset PSCI (including PSD and PSCI not fulfilling criteria for dementia) is still in lack, while the prevalence of delayed-onset PSD varies largely from 4.4 to 23.9% according to a systematic review [6]. Though it seems lower comparing with early-onset PSD, the risk of dementia is still 1.6 to 10.3 times higher than that in stroke-free population. Study of these group of patients is important because there might be different mechanism from early-onset PSCI, which leads to a different optimal treatment plan.

Studies showed that early-onset PSCI mainly resulted from acute stroke lesions [7], while delayed-onset PSCI was found specifically associated with presence of severe cerebral small vessel diseases (CSVD) at baseline [6, 8]. Nevertheless, recent longitudinal studies have further indicated that the primary mechanism may be cerebral secondary degeneration (CSVD progression presented as increased white matter hyperintensity and cerebral atrophy) induced by acute stroke apart from baseline co-existent CSVD [9–11]. However, no interventional researches have been conducted to testify the role of secondary degeneration in its mechanism.

DL-3-n-butylphthalide (NBP) is a synthetic compound based on L-3-n-butylphthalide which is initially isolated from seeds of *Apium graveolens*. In 2005, the State Food and Drug Administration of China approved NBP as a therapeutic drug for ischemic stroke as it could significantly improve neurological function and exhibit good safety and tolerability, making it the first-class and recommended drugs in the Chinese guidelines of acute ischemic stroke. There is only one commercial dosage form of NBP in China: 100 mg for one soft capsule and 24 capsules in a bottle. Based on evidences of the multi-center phase 2 and 3 randomized controlled clinical trials [12, 13], oral administration of 200 mg NBP soft capsules, three times daily showed greatest effectiveness and good safety in the neurological recovery after acute ischemic stroke, thus has become the officially recommended dose. Massive evidences have found that NBP could achieve its protective function through multitargeted actions: antiplatelet aggregation and antithrombotic effects, protection of mitochondria, anti-oxidant, anti-apoptosis, improvement of cerebral microcirculation, promotion of neurogenesis and poststroke brain tissue recovery, etc. [14] Clinical researches have also testified that combined treatment of NBP might improve cerebral hypoperfusion in the patients with carotid artery atherosclerotic stenosis [15] and may improve revascularization and inhibit inflammation in acute stroke patients (reflected by blood biomarkers) [16–18]. What’s more, recently, both animal and clinical researches have validated that NBP could alleviate ischemia-induced cognitive deficits and one target could be white matter degeneration [19–22]. Hence, based on possible mechanisms of delayed-onset PSCI and NBP’s action targets, we hypothesized that NBP may have preventive efficacy for delayed-onset PSCI (including PSD and PSCI not fulfilling criteria for dementia) and then designed the present study.

## Methods

### Design

**Fig. 1:**
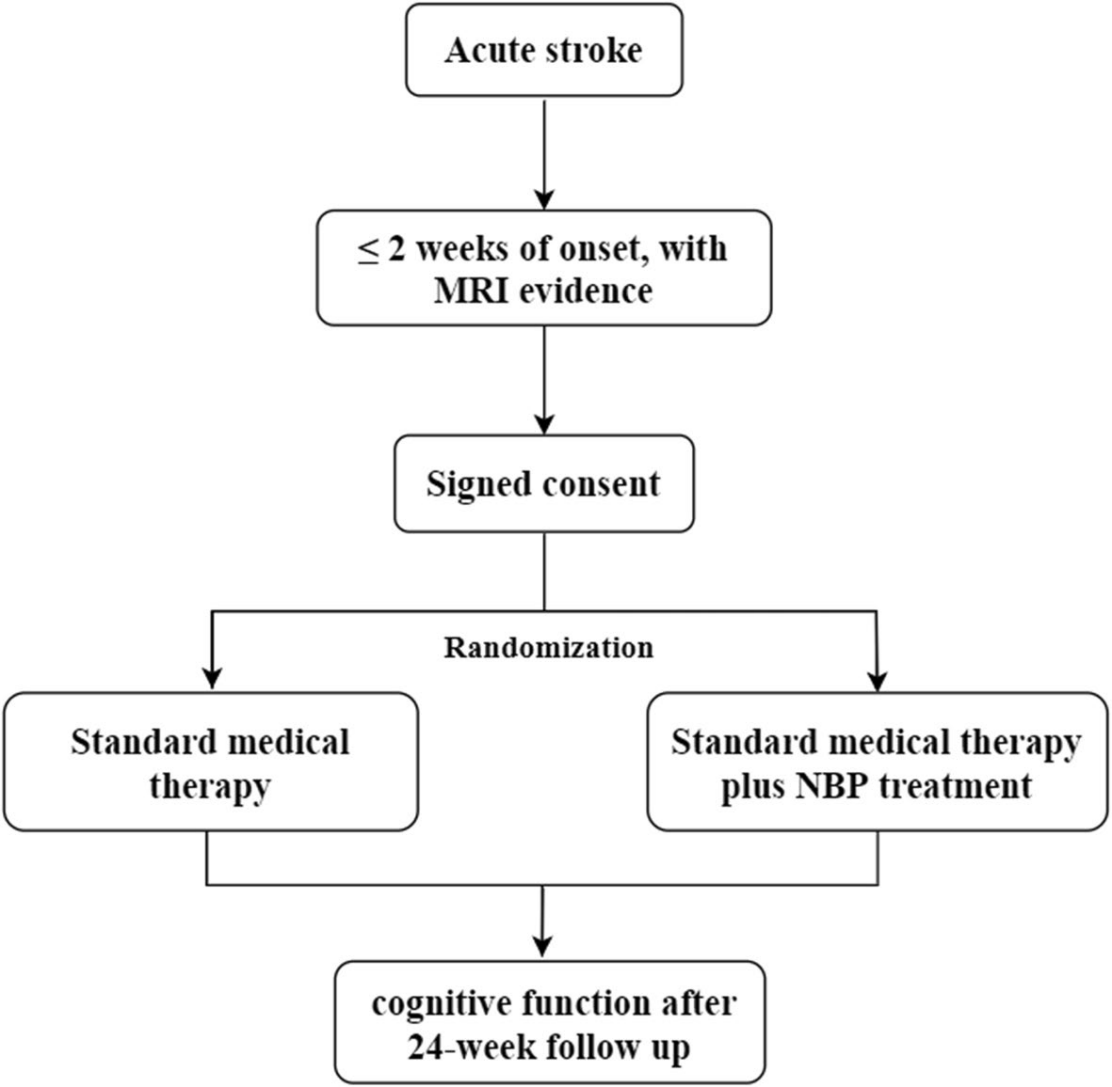
Flow chart of End-PSCI trial. This is the flow chart of End-PSCI trial. Hospital patients with acute cerebral infarction (within 2 weeks of onset and with MRI evidences) will be randomized into either standard medical therapy group or standard medical therapy combined NBP treatment group. Cognitive function will be tested during the 24-week follow-up. MRI: magnetic resonance imaging, NBP: DL-3-n-butylphthalide

Effects of NBP on Delayed-onset Post Stroke Cognitive Impairment (End-PSCI) is a prospective, parallel-group, open-label, multicenter, randomized controlled trial with blinded outcome assessment. Hospital patients within 2 weeks of cerebral infarction onset will be randomized into either standard medical therapy group or standard medical therapy combined NBP treatment group. A set of validated cognitive tests and blood samples collection will be implemented at acute phase (within 2 weeks of onset), 6 weeks, 12 weeks and 24 weeks after stroke. Brain magnetic resonance imaging (MRI) including T1, T2 and T2 Fluid-attenuated inversion recovery imaging (most are clinical routine scans with some high resolution, isotropic scans) will also be performed at acute phase and 24 weeks after stroke. An independent Data and Safety Monitoring Board (DSMB) will regularly monitor safety during the study. The trial has been approved by the Institutional Review Board/Ethics Committee in the First Affiliated Hospital of Xi’an Jiaotong University (approval number: XJTU1AF 2020LSK-032). Figure 1 this study is ongoing (recruitment not completed) at the time of submission.

### Patient population

Hospital acute cerebral infarction patients confirmed by MRI will be enrolled and randomized within 2 weeks of onset. The detailed inclusion and exclusion criteria are listed in Table 1.

**Table 1:** Inclusion and exclusion criteria of End-PSCI

**Inclusion criteria**	
1. Age ranges from 50 to 75 years (50 and 75 also included), no gender limitation.	
2. Meet the diagnostic criteria of Chinese guidelines for diagnosis and treatment of acute ischemic stroke 2018^a^ [23].	
3. Brain MRI provides evidences for the diagnosis of acute cerebral infarction.	
4. No disturbance of consciousness, evident aphasia or severe paralysis.	
5. Educational level should be primary school or above, with capability of finishing cognitive tests.	
6. Signed written informed consent.	
**Exclusion criteria**	
1. Patients treated by intravenous or arterial thrombolysis or endovascular interventional therapy.	
2. Patients with secondary hemorrhage or hematoma after cerebral infarction.	
3. Patients with pre-stroke cognitive impairment.	
4. Patients with other severe central nervous systematic diseases: Alzheimer’s disease, Parkinson’s disease, dementia with Lewy bodies, frontotemporal dementia, neuromyelitis optica, epilepsy, central nervous system infection, cerebral injury, cerebral tumor, etc.	
5. Patients with mental disorders diagnosed by the Diagnostic and Statistical Manual of Mental Disorders—Fourth Edition Text Revision (DSM-IV-TR) [24], including schizophrenia, bipolar affective disorder, major depressive disorder or delirium, etc.	
6. Patients are taking cholinesterase inhibitors, Memantine or NBP.	
7. Patients with uncorrectable visual or hearing disturbances severe enough to preclude cognitive tests.	
8. Patients with unstable or severe cardiovascular, respiratory, gastrointestinal, hepatic, renal or hematopoietic system disease.	
9. Unable to perform MRI tests.	
10. Patients who are unwilling or unable to comply with the protocol or cannot/will not cooperate fully with the investigator.	

^a^The following should all be met: 1) Acute onset; 2) With focal neurological function defects (e.g. one side limb weakness, numbness of one side of the face or language disorders), a few with general neurological function defects; 3) Responsible lesions on imaging or symptoms/signs lasted more than 24 h; 4) Excluded non-vascular etiology; 5) Brain CT/MRI excluded cerebral hemorrhage

### Randomization

Eligible patients will be randomly allocated to receive either standard medical therapy or standard medical therapy plus NBP treatment (1:1) using block randomization in blocks of eight. The randomization code list will be generated by an independent statistician and directly allocated to the main investigators in every center so that the independent and specialized cognitive evaluators and the diagnostic group will be blinded.

### Interventions

**Fig. 2:**
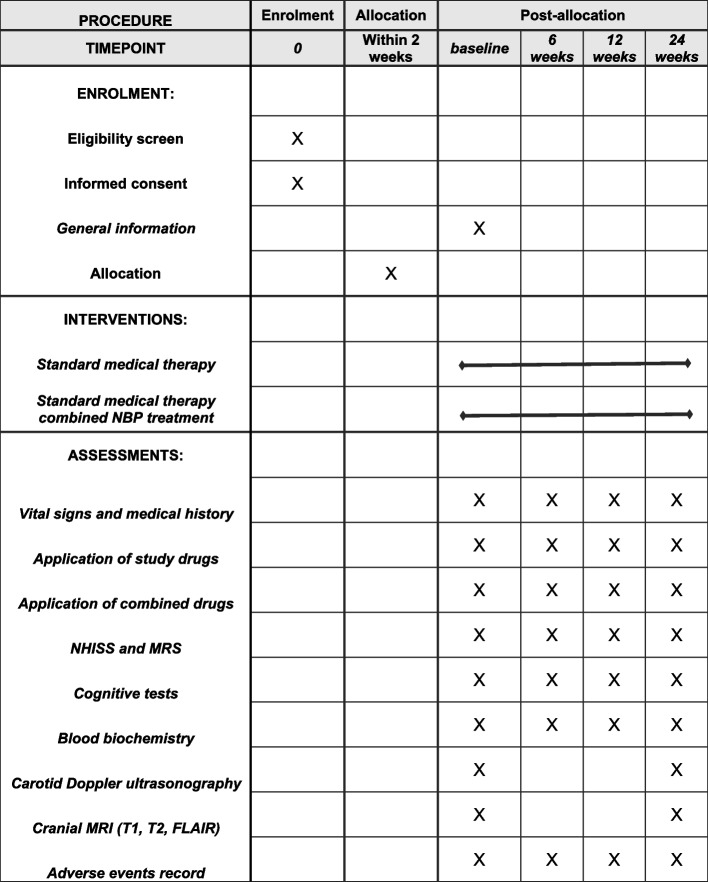
Schedule of End-PSCI. This is the schedule of End-PSCI trial. After enrolment and allocation, general information will be recorded at baseline. The treatment of standard medical therapy and standard medical therapy combined NBP treatment will last for 24 weeks after stroke. For every follow-up visit (baseline, 6 weeks, 12 weeks and 24 weeks after stroke), vital signs and medical history, application of study drugs, application of combined drugs, NHISS and MRS scores, a set of validated cognitive tests, blood samples collection and adverse events record will be implemented. Carotid Doppler ultrasonography and brain MRI including T1, T2 and T2 Fluid-attenuated inversion recovery imaging will also be performed at baseline and 24 weeks after stroke. NHISS: National Institutes of Health Stroke Scale; MRS: Modified Rankin Scale; MRI: Magnetic Resonance Imaging; FLAIR: axial fluid-attenuated inversion recovery

Standard medical therapy will be implemented by attending physicians according to the Chinese official guidelines. This usually involves use of antiplatelet drugs, anti-hypertension drugs, antidiabetic drugs or lipid-lowering drugs etc. Standard medical therapy plus NBP treatment group will add NBP of 200 mg (oral), three times per day for 24 weeks on the basis of conventional treatment. The dose of NBP treatment is consistent with the officially recommended treatment plan for acute ischemic stroke as well as all other NBP clinical trials targeting cognitive impairment [21]. Appropriate number of NBP will be prescribed to ensure they are just enough for the time until next visit and compliance will be assessed by counting empty bottles of NBP at every visit. Blood biochemical tests will be performed at every visit for regular examination and adverse effect monitoring. Previous studies reported a few cases of mild elevation of transaminase after NBP treatment. If this happens, medication will be withdrawn immediately and the condition will be monitored (and treated if necessary) until it returns to normal. This will be treated as drop-out. The schedule of End-PSCI is showed in Fig. 2**.**

Notably, long-term use of two types of drugs should be avoided in all enrolled patients. One is that may influence cerebral microcirculation or metabolism such as Nimodipine, Idebenone, Oxiracetam and some traditional Chinese medicine that have blood activating and stasis removing effects. Another type is that may influence cognitive function such as cholinesterase inhibitors, Memantine and antipsychotic drugs. If happens, this will be treated as drop-out.

### Primary outcome

The primary outcome is the difference of incidence of delayed-onset PSCI between two groups after 24 weeks. We here define delayed-onset PSCI as cognitive impairment including mild cognitive impairment and dementia developed in 3–6 months after stroke, without early-onset PSCI. A group of trained neurologists who are blinded from allocation will make the final diagnosis at 24 weeks in the main center based on the Vascular Behavioral and Cognitive Disorders (VASCOG) criterion [25]. Mild cognitive impairment will be diagnosed based on 1) Concerns of a patient, knowledgeable informant, or a clinician of mild levels of decline in ≥1 cognitive domains from a previous level of cognitive functioning, and 2) Evidence of modest deficits in ≥1 cognitive domains on the neuropsychological testing (fall between 1 and 2 standard deviation below the mean (or between the third to 16th percentiles for test scores not normally distributed) of people of similar age, sex, education, and sociocultural background). And The cognitive deficits are not sufficient to interfere with independence (instrumental activities of daily living are preserved). Dementia will be diagnosed based on 1) Concerns of a patient, knowledgeable informant, or a clinician of significant levels of decline in ≥1 cognitive domains from a previous level of cognitive functioning, and 2) Clear and significant deficits in ≥1 cognitive domains on the neuropsychological testing (fall ≥2 standard deviation below the mean (or below the third percentile) of people of similar age, sex, education, and sociocultural background). And the cognitive deficits are sufficient to interfere with independence (e.g. at a minimum requiring assistance with instrumental activities of daily living).

### Secondary outcomes

1. The difference of white matter degeneration between two groups after 24 weeks. The degeneration will be reflected by changes of visual scale scores (modified Scheltens Visual Scale) [26] and white matter hyperintensity (WMH) volume from baseline to 24 weeks.
2. The difference of cognitive score changes from baseline to 24 weeks between two groups. Cognitive testing includes Montreal Cognitive Assessment, Beijing Version (MoCA-BJ) [27], Alzheimer’s Disease Assessment Scale-Cognitive Subscale, Chinese version (ADAS-Cog-C) [28], Hamilton depression Rating scale (HAMD-17) [29] and Activities of Daily Living Scale (ADL), Chinese version [30].
3. The difference of prevalence of early-onset PSCI between two groups after 24 weeks. Early-onset PSCI is defined as cognitive impairment including mild cognitive impairment and dementia developed within 3 months after stroke and persistently existed at least for 3 months.

### Data monitoring body

A Steering Committee chaired by Dr. Qu from the First Affiliated Hospital of Xi’an Jiaotong University will take charge of overall design, co-ordination, financial management and publications.

The independent DSMB, consisting of experienced neurologists, academic members and an independent statistician, will meet every 6 months to assess progression, data quality, safety and efficacy of the trial. Recommendations on whether to continue or stop the trial will be given to the steering committee. All adverse events will be recorded and managed properly until they are resolved or reach stability.

### Sample size

Previous studies showed that the prevalence of delayed-onset PSD ranged from 4.4 to 23.9% [6]. We chose 4.4, 10.0 and 23.9% with the hypothesized effect size of 20, 30, and 50% reduction for NBP to do the calculation [21]. Considering the feasibility, 4.4% of the incident rate and reduction of 50% were used in the end, then 1200 cases for per group could meet the statistical requirement. A drop-out rate of 20% was also considered, then 3000 cases in total were required.

### Statistical analyses

A customized online database will be used to manage data and SPSS version 25.0 will be used for all analyses. Group t-test, Mann-Whitney u test or χ2 test will be conducted for comparisons between two groups according to different types of variables. Occurrence of delayed-onset and early-onset PSCI will be analyzed as categorical data (yes or no). χ2 test will be used to compare the incidence between two groups and multivariate logistic regression will be used to adjust confounders. Paired sample t-tests or Wilcoxon matched-pairs signed-ranks test will be used to compare WMH degree (visual scale scores and WMH volume) and total cognitive scores at different time point within each group. Changes of WMH degree (indicating degeneration) and total cognitive scores from baseline to 24 weeks will be compared between two groups using Group t-test or Mann-Whitney u test and multivariate linear regression will be used to explore the contributing factors. Changes of cognitive scores will further be dichotomized as improved and unimproved group to do the analysis, improvement is defined as score change < 0 for Alzheimer’s Disease Assessment Scale-Cognitive subscale or > 0 for Montreal cognitive assessment scale. Furthermore, Kaplan–Meier analysis and cox proportional hazards analysis will also be performed to compare the incident rate of any cognitive impairment between two groups and explore contributing factors. Two-sided value of *p* < 0.05 will be considered significant.

Outcomes will be measured both for intention-to-treat population (main analysis population) and the per protocol population. The intention-to-treat population consist of those who have a complete baseline assessment as well as at least one follow-up assessment for the primary outcome variables. Missing values of cognitive scores will be replaced by last observation carried forward method. The per protocol population consist of those who complete the 24-week follow-up with no major protocol violations.

### Study organization

The study organization includes: (1) Steering Committee; (2) DSMB; (3) Executive Committee. The Executive Committee includes principal investigators (usually the attending doctors) in every center, independent and specialized cognitive evaluators in every center and senior neurologists from the main center who will make the final diagnosis. The principal investigators are responsible for enrolment, allocation and follow-up visit. The evaluators will perform cognitive testing in a separate examination room without knowing the patients’ any information except name and educational level. The diagnostic group will make the final diagnosis based on clinical information (not including allocation and medication administration record). All investigators will undergo standard training courses on study protocol and confidentiality. The independent cognitive evaluators will also be trained not to ask or answer any questions about the patients’ information and be assessed for consistency before getting certification for cognitive tests.

## Discussion

End-PSCI is designed to assess the preventive effects of NBP on delayed-onset PSCI. To our knowledge, this is the first large randomized controlled trial (RCT) for NBP in PSCI. Previously, a small RCT showed combined NBP treatment could significantly improve the cognitive function 1 month after stroke along with lower serum inflammatory factors [31]. While in this study, cognition was tested at a very short time after stroke which was believed not accurate to reflect the true cognitive function after stroke because of the transient delirium. In 2016, Jia, et al. [21] found NBP significantly increased cognitive scores compared with placebo in patients with subcortical vascular cognitive impairment without dementia in China, after treatment with NBP 200 mg three times daily for 24 weeks. Subcortical vascular cognitive impairment closely associates with CSVD in which white matter injuries play an important role. However, Jia’s trial couldn’t explore the role of white matter injuries in its mechanism due to lack of imaging examination and white-matter-related fluid biomarker tests. In End-PSCI trial, we preserve blood samples at every visit and MRI data at baseline and end time, so that both direct and indirect indications of white matter injuries can be assessed before and after intervention. By doing this, we will provide evidences for NBP’s action on white matter and testify secondary degeneration theory in delayed-onset PSCI.

Treatment duration of NBP is not officially restricted. It varied from 4 weeks to 2 years in different studies targeting cognitive impairment and all showed favorable results [21, 31, 32]. Since this is a preventive study and we have defined delayed-onset PSCI as cognitive impairment occurred in 3–6 months post stroke, we then decide 6 months (24 weeks) of treatment might be appropriate.

Notably, two types of treatment should be avoided in our study as the first type may act on some same targets with NBP and the second type may influence the cognitive outcomes. The avoidance of first type of treatments will not interfere with the optimal treatments because these are not recommended in routine treatment of cerebral infarction according to the Chinese official guidelines. The second type of treatments are only evidentially recommended in patients with confirmed diagnosis of dementia or mental illness. For patients with confirmed or suspicious diagnosis of newly dementia or mental illness, treatment plan will be determined by doctors and negotiated with patients or caregivers according to patients’ condition. If these treatments are applied, patients will be treated as drop-out, or on the contrary, continue in the study. Confirmed early-onset PSCI patients will continue in the study unless they take medications that should be avoided during the study, which will be recorded and treated as drop-out. With proper informed consent, the treatment plan will be negotiated with these patients or caregivers to decide whether they will continue in the study. These patients will be given routine treatments immediately after the last visit if necessary. Notably, the confirmation of early-onset PSCI will be made at least 3 months after the symptom, and the visit plan is at about 1, 3 and 6 months, which will not significantly delay the treatment.

One drawback of End-PSCI trial is the open-label nature. The main aim of the present study is to investigate the preventive effects of NBP on delayed-onset PSCI, and the primary outcome is defined as the difference of incidence of delayed-onset PSCI between study group and control group. However, due to the lower incidence of delayed-onset PSCI, the sample size would be about 3000 patients with acute cerebral infarction from the calculation based on the primary outcome, therefore will cost too much to provide placebo medicines. Hence, we design this study as open label. To avoid bias, efforts have been made by blinded outcome assessment that allocation will be blinded from all cognitive evaluators and senior neurologists who make the final diagnosis.

## Data Availability

The datasets generated and/or analyzed during the current study are not publicly available because these clinical datasets contain direct and indirect identifiers, but are available from the corresponding author on reasonable request.

## Abbreviations

PSCI: Post stroke cognitive impairment
PSD: Post stroke dementia
CSVD: Cerebral small vessel diseases
NBP: DL-3-n-butylphthalide
End-PSCI: Effects of NBP on Delayed-onset Post Stroke Cognitive Impairment
DSMB: Data and Safety Monitoring Board
MRI: Magnetic Resonance imaging
VASCOG: Vascular Behavioral and Cognitive Disorders
WMH: White matter hyperintensity
RCT: Randomized controlled trial
